# Angiotensin II, miR-34a, and AGTRAP crosstalk in arterial smooth muscle cells

**DOI:** 10.1007/s11357-025-02018-5

**Published:** 2025-11-26

**Authors:** Maria Cristina Florio, Sara Sileno, Liqun Jiang, Marco D’Agostino, Sunayana Begum Syed, Laura Monteonofrio, Mirko Baranzini, Chiara Prampolini, Federica Macrì, Manuel Casaburo, Nadia Fanotti, Stefania Castiglione, Robert E. Monticone, Bruce Ziman, Richard Telljohann, Mingyi Wang, Julie A. Mattison, Christopher H. Morrell, Angela Raucci, Edward G. Lakatta, Alessandra Magenta, Maurizio Colognesi-Capogrossi

**Affiliations:** 1 Laboratory of Cardiovascular Science, National Institute On Aging, National Institutes of Health, Baltimore, MD USA; 2 Laboratory of Molecular Regenerative Medicine, Istituto Dermopatico Dell’Immacolata (IDI-IRCCS), Rome, Italy; 3 Istituto Nazionale Tumori Regina Elena IRCSS, Rome, Italy; 4 Unit of Experimental Cardio-Oncology and Cardiovascular Aging, Centro Cardiologico Monzino IRCCS, Milan, Italy; 5 Animal Facility, Centro Cardiologico Monzino IRCCS, Milan, Italy; 6 Translational Gerontology Branch, National Institute On Aging, NIH Animal Center, Dickerson, MD USA; 7 Institute of Translational Pharmacology (IFT), National Research Council (CNR), Rome, Italy; 8 Division of Cardiology, Johns Hopkins Bayview Medical Center, Johns Hopkins University School of Medicine, 301 Building, Baltimore, MD USA

**Keywords:** AGTRAP, SIRT1, Aging, Arterial aging, MFGE8, MiR-34a

## Abstract

Arterial aging is associated with enhanced angiotensin II (Ang II) signaling via Ang II type 1 receptor (AT1R) and with microRNA-34a (miR-34a) increased expression. AT1R-associated protein (ATRAP/Agtrap) binds to AT1R, promotes its internalization, and inhibits Ang II signaling. This study addresses the hypothesis that miR-34a targets ATRAP/Agtrap and enhances Ang II pro-inflammatory signaling via AT1R in arterial vascular smooth muscle cells (VSMC). Our results show that miR-34a exhibits an age-associated increase in Rhesus monkey’s common carotid artery and rat aorta. Further, AGTRAP protein expression is lower in old rat VSMC and in old mice aorta. Ang II enhances miR-34a in old rat VSMC and human aortic smooth muscle cells (HASMC), and inhibits AGTRAP and sirtuin 1 (SIRT1) mRNA/protein expression. In miR-34a-overexpressing HASMC, AGTRAP and SIRT1 mRNA/protein decrease, and these effects are rescued by AGTRAP forced expression. Moreover, miR-34a directly targets AGTRAP and AGTRAP downmodulation further enhances miR-34a expression decreasing SIRT1 in HASMC. Finally, Ang II and miR-34a induce the upregulation of pro-inflammatory genes, interleukin (IL)-6, cyclooxygenase 2 (COX2), monocyte chemoattractant protein-1 (MCP-1), and milk fat globule-epidermal growth factor 8 (MFGE8) in HASMC, and this effect is abolished by AGTRAP forced expression. In conclusion, Ang II upregulates miR-34a, activating a negative feedback loop on AGTRAP that reinforces Ang II signaling. The age-associated AGTRAP downmodulation in central arteries and VSMC underlies a potential miR-34a/AGTRAP role in vascular aging.

**Supplementary Information:**

The online version contains supplementary material available at 10.1007/s11357-025-02018-5.

## Introduction

Arterial aging is associated with intima-media thickening, leading to increased vascular stiffness; endothelial dysfunction, enhanced arterial vascular smooth muscle cells (VSMC) migration and proliferation, and extracellular matrix deposition [1, 2] account for the age-associated increased intima-media thickness and its functional sequelae. It is noteworthy that, in addition to aging per se, intima-media thickening is also associated with other conditions, including atherosclerosis, hypertension, and diabetes that are more common in the elderly population [3–5].

The Renin–Angiotensin–Aldosterone System (RAAS) and, specifically, angiotensin II (Ang II), plays a major role in the chronic low-grade, “sterile” pro-inflammatory state that underlies arterial aging [6], and the effect of Ang II, at least in part, is mediated by milk fat globule-epidermal growth factor 8 (MFGE8), an inflammatory protein that is induced by Ang II and exhibits an age-associated increase in the arterial wall [7, 8]. The mechanisms for the activation of the pro-inflammatory RAAS pathway have been partially elucidated, and it has been shown that an age-associated increased expression of angiotensin-converting enzyme (ACE), responsible for converting angiotensin I (Ang I) into Ang II, occurs. Increased Ang II is paralleled by a decreased expression of ACE2, which converts the pro-inflammatory Ang II into the anti-inflammatory Ang (1–7), as well as by an increase of Ang II type 1 receptor (AT1R) expression and a decrease of AT2R and Mas receptor [6, 9, 10]. The net effect of these age-associated changes is increased signaling via the pro-inflammatory and pro-fibrotic AT1R and decreased signaling via the anti-inflammatory and anti-fibrotic AT2R and MAS receptor. An important inhibitor of Ang II signaling is AT1R-associated protein (ATRAP/Agtrap), which was initially shown to diminish AT1R-mediated activation of phospholipase Ca^2+^ signaling by enhancing AT1R internalization [11–13] and by disrupting Calcium-modulated Cyclophilin Ligand (CAML) activation of nuclear factor of activated T cells (NFAT) [14, 15]. Further, it was established that AGTRAP-knockout (KO) mice exhibit a decreased lifespan and enhanced renal fibrosis [16] and that AGTRAP overexpression inhibits Ang II–induced hypertension [17] and inflammatory vascular remodeling [18]. The functional role of AGTRAP in VSMC, the effect of aging on AGTRAP expression in the arterial wall, and the mechanisms that modulate AGTRAP expression are still poorly characterized.

Multiple microRNAs (miRNAs) are modulated during aging across various tissues and their dysregulation appears to contribute to the aging phenotype [19, 20]. These miRNAs influence fundamental hallmarks of aging; for example, by modulating DNA damage responses, cellular senescence, inflammation, and metabolic pathways. Several age-associated miRNAs are associated with cardiovascular aging [21]; among them, miR-34a increases in the aorta of 21-month-old vs 2.5-month-old mice [22] and it induces arterial calcifications, a phenomenon closely linked to arterial aging and atherosclerosis, by downregulating SIRT1 and AXL receptor tyrosine kinase [23]. In vascular cells, miR-34a induces cellular senescence and the pro-inflammatory senescence-associated secretory phenotype (SASP). Further, miR-34a directly targets longevity and survival genes such as SIRT1 and Bcl-2 [24, 25], thereby promoting endothelial dysfunction and VSMC aging. There is a significant overlap between Ang II and miR-34a effects, and the interaction between Ang II and miR-34a has been partially examined in the case of abdominal aortic aneurysms. Ang II enhances miR-34a expression [26], and miR-34a deletion inhibits Ang II–induced aneurysm formation [26, 27]. The protective mechanism of miR-34a deletion on Ang II–induced aneurysm formation has not been elucidated, and the mechanisms underlying the interaction between miR-34a and Ang II signaling are still unknown.

In summary, miR-34a is a well-established and important “aging-microRNA”; Ang II increases miR-34a expression, and it has been demonstrated that enhanced Ang II signaling is a key determinant of vascular aging.

In light of the role of AGTRAP as an inhibitor of Ang II signaling via AT1R, we aimed to investigate the effect of miR-34a on AGTRAP and the impact of such interaction on Ang II-dependent inflammatory mechanisms.

## Materials and methods

### Animal models

All experiments with rats and non-human primates (NHPs) were conducted according to the NIH Guide for Care and Use of Laboratory Animals and approved by the National Institute on Aging (NIA) Intramural Research Program Animal Care and Use Committee. All procedures were conducted in accordance with the Guide for the Care and Use of Laboratory Animals and approved by the NIA/NIH Intramural Research Program’s Animal Care and Use Committee.

Experiments with mice were performed in conformity with the guidelines from Directive 2010/63/EU of the European Parliament on the protection of animals used for scientific purposes and in accordance with the experimental protocol approved by the Animal Welfare Organization (OPBA) of the Italian National Ministry of Health and Cogentech (824/2020-PR).

miR-34a^−/−^ (miR-34a KO) mouse line was purchased from the Jackson Laboratory (Bar Harbor, ME, USA). JAX™ C57BL/6 J male wild-type mice miR-34a^+/+^ (WT) were purchased from Charles River Laboratories (Calco, Italy). Mice were housed in standard cages and fed a normal chow diet. Young (10 weeks) and old (18 months) male miR-34a KO and WT mice were generated, anesthetized with an intraperitoneal injection of ketamine (100 mg/kg), and perfused with physiologic saline. Aortas were isolated and processed for immunohistochemistry analysis.

Male Fisher 344X Brown Norway rats, 8- and 30-months-old, were obtained from the Aged Animal Colony (NIA/NIH) and were maintained at 26 °C in a temperature-controlled room, on a 12-h light/dark cycle, with free access to food and water. Rats were sacrificed by exsanguination from the thoracic aorta under deep anesthesia with pentobarbital (150 mg/kg). The aorta was excised and used for gene expression analysis or isolation of VSMC.

The rhesus monkeys (*Macaca mulatta*) were maintained at the NIH Animal Center (Poolesville, MD). NHP were provided a Western-type semi-purified diet with 20% protein, 49% carbohydrate, and 31% fat. Monkeys were fed twice daily with the goal of weight maintenance, and water was available ad libitum. They were housed in standard primate caging with a controlled temperature (25.5 ± 0.5 °C) and humidity (60 ± 20%), and a 12-h light–dark cycle. Common carotid arteries were obtained from monkeys (*n* = 10) 10.8–28.1 years old, and kidney samples were obtained from monkeys (*n* = 20) 5.8–30.5 years old. At the time of euthanasia, monkeys were anesthetized with ketamine (7–10 mg/kg, IM) followed by an overdose of pentobarbital (50 mg/kg, IV). Common carotid arteries and kidney biopsies were immediately harvested, flash frozen, and stored at − 80 °C until analyzed.

### Adventitia and media separation and vascular smooth muscle cell isolation

Adventitia and media were separated, and VSMC were isolated from the rats’ aorta as previously described [28]. Thoracic aorta was removed and washed in HBSS solution and then transferred in a sterile hood into a clean 100-mm dish with fresh HBSS. Fat was gently removed under a microscope and incubated in a 35-mm dish containing DMEM and 2 mg/mL collagenase I for 30 min at 37 °C.

The tissue was washed in fresh HBSS and cut longitudinally. The inner part of the aorta was gently scraped to remove the endothelium layer, and then the adventitial layer and media layer containing the VSMC were separated and used for the analysis of miR-34a expression. The successful separation of media (smooth muscle layer) from adventitia was confirmed by α-smooth muscle actin (α-SMA) staining. For VSMC isolation, the media layer was incubated for 90 min at 37 °C with DMEM, 2 mg/mL collagenase II, and 0.5 mg/mL elastase. After incubation, an equal volume of DMEM containing 10% FBS was added to the dish and mixed for proper dissociation. Cells were filtered through a cell strainer (70 µm) and centrifuged at 1500 rpm for 5 min. The cell pellet was re-suspended in DMEM containing 20% FBS and seeded on 60-mm dishes. VSMC were used for the described experiment up to passage 4–6. VSMC were characterized and checked for cross-contamination by immunocytochemistry.

### Human aortic smooth muscle cell culture

Human aortic smooth muscle cells (HASMC) from a 22-year-old Caucasian man were purchased from Lonza (Basel, Switzerland) and cultured in SmGM-2 medium (Lonza).

### Angiotensin II and valsartan treatment

Rat aorta VSMC were treated with human Ang II (Ang II; Sigma Aldrich St. Louis, MA, USA) at 100 nM for 48 h after 24 h starvation in 1% FBS DMEM. HASMC were treated for 24 h with Ang II (1 µM), after 4 h starvation in 1% FBS SmGM-2.

Valsartan (Sigma Aldrich, St. Louis, MA, USA) was dissolved in DMSO. HASMC were serum-starved for 3 h in 1% FBS SmGM-2. The cells were then treated with Valsartan (1 µM) for 1 h, followed by treatment with Ang II (1 µM) for 24 h.

### miR-34a and AGTRAP over-expression

miR-34a overexpression in HASMC was achieved by lentiviral infection using pre-miR-34a lentiviral supernatants (pMIRH34a, System Biosciences, SBI) or a control sequence (miR-scramble). Lentiviral supernatants were produced as previously described, using standard procedures [29].

The plasmid pReceiver-Lv105-AGTRAP human (NM_020350.4) transcript variant 1 (Gene Copoeia) was used for AGTRAP overexpression.

HASMC co-expressing miR-34a and AGTRAP were produced by co-infection of cells with both lentiviral supernatants for 2 h. Control cells were co-infected with the miR-scramble sequence and AGTRAP backbone vector (pReceiver-Lv105) supernatants. Single infections with miR-34a or AGTRAP were performed together with pReceiver-Lv105 or miR-scramble viruses, respectively, for 2 h. Cells were collected 72 h after infection.

### AGTRAP inhibition

MISSION shRNA lentiviral control and AGTRAP-specific construct TRCN0000060811 were purchased from Sigma. The target sequence of the AGTRAP transcript variant 1, reference sequence: NM_020350 was CGTAGTGCCTACCAGACGATT. HASMC were infected for 2 h with lentiviral supernatants and then were allowed to recover in complete fresh medium for an additional 24 h. Afterward, puromycin-containing medium (0.5 µg/mL, Sigma Aldrich St. Louis, MA, USA) was added to the cells.

### Luciferase assay

We found by in silico analysis (TargetScan V8.0) that AGTRAP 3′UTR contains the miR-34a seed sequence.

HEK293 cells were plated in 12-well plates and were transfected with either 0.5 g of the full-length 3′-UTR luciferase construct pEZX-MT06-3′UTR-AGTRAP (NM_020350.4) or the mutated one pEZX-MT06-3′UTR-AGTRAP1 mut, in which the seed sequence ACTGCC spanning from position 451–457 nt of the 3′UTR was mutated to AGTCCG. The position in the latest version of the 3′UTR of Human AGTRAP ENST00000376627.2 is 584–590 of 3′UTR. The constructs were co-transfected either with 0.25 µg of pre-miR-34a (PMIRH34aPA-1 Human pre-miR-34a System Biosciences (SBI), Palo Alto, CA, USA) or 0.25µg scramble control.

Cellular extracts were tested with Dual Luciferase Assay (Promega, Madison, WI, USA) according to the manufacturer’s instructions, using an EnSight plate reader (PerkinElmer, Waltham, MA, USA). Values were normalized according to the renilla luciferase activity.

### RNA purification and reverse transcription quantitative PCR

Total RNA, including miRNA, was isolated using TRIzol® reagent (Invitrogen; Thermo Fisher Scientific, Inc., Waltham, MA, USA) according to the manufacturer’s instructions. For microRNA evaluation, reverse transcription was performed using the Taqman microRNA reverse transcription kit (Applied Biosystems™, Thermo Fisher Scientific, Inc.) in conjunction with miRNA-specific primers. miR-34a expression levels were normalized to hsa-miR-16 expression (HASMC) or U6 snRNA (VSMC). Reverse Transcription quantitative PCR (RT-qPCR) was performed using TaqMan™ Universal PCR Master Mix II, no AmpErase UNG (Applied Biosystems, ThermoFisher Scientific, Inc.). The same miRNA probes were used for all species.

For gene expression evaluation, the reverse transcription was generated by the SuperScript First-Strand Synthesis System (Invitrogen, Paisley, U.K.) and RT-qPCR was performed with the SYBR GREEN RT-qPCR method using the QuantStudio 5 Real-Time PCR Instrument (Applied Biosystem, Thermo Fisher Scientific, Inc.). mRNA expression was normalized for the nuclear small subunit rRNA (18S). Relative RNA expression was calculated using the comparative Ct method (2 − ΔΔCt). The primer sequences used in the present study are listed in Table 1.

**Table 1:** Oligonucleotide-specific primers for gene expression

Gene	Forward Primer (5′–3′)	Reverse Primer (5′–3′)
SIRT1	AAATGCTGGCCTAATAGAGTGG	TGGCAAAAACAGATACTGATTACC
AGTRAP	TGCTGTGAACCTGAAGGTGA	TGAAGTTGGCCCAGGCATAG
COX2	CTTCACGCATCAGTTTTTCAAG	TCACCGTAAATATGATTTAAGTCCAC
IL-6	GATGAGTACAAAAGTCCTGATCCA	CTGCAGCCACTGGTTCTGT
MCP1	AGTCTCTGCCGCCCTTCT	GTGACTGGGGCATTGATTG
MFGE8	GACAAGCAGGGCAACTTCAA	GATGATGCCTGTCACCTCCT
rRNA 18S	CGAGCCGCCTGGATACC	CATGGCCTCAGTTCCGAAAA

### Immunohistochemistry

Mouse distal thoracic aortas were fixed in 10% formalin and embedded in paraffin. Six µm sections were de-paraffinized, re-hydrated, boiled in Dako Target Retrieval Solution Citrate pH 9 (Aligent Technologies, Santa Clara, CA, USA), and incubated in 3% H_2_O_2_ (Sigma-Aldrich). For AGTRAP staining, slides were blocked in 5% goat serum-PBS-T for 45 min at room temperature. Primary antibody against AGTRAP (10 µg/mL, Thermo Fisher Scientific, Waltham, MA, USA) was dissolved in 1% goat serum-PBS-T and incubated overnight at 4 °C.

For angiotensinogen staining, slides were blocked in PBS-T-5% BSA for 1 h at room temperature. The primary antibody against angiotensinogen (Abcam, Cambridge, UK) was dissolved in 1% BSA-PBS-T, and sections were incubated overnight at 4 °C in a humidified chamber. Adjacent sections, in which the tissue was incubated with 1% goat serum PBS-T or 1% BSA-PBS-T without the primary antibody, were used as negative controls to test the specificity of the staining. Then, sections were incubated with biotin-conjugated goat anti-rabbit antibody (Vector Laboratories, Burlingame, CA, USA) and with horseradish peroxidase (HRP)-conjugated streptavidin (ABC kit; PK-6100, Vector Laboratories) at room temperature. Immunoreactions were revealed using 3.3′-Diaminobenzidine (90 s exposure; ImmPACT DAB substrate, SK-4105, Vector Laboratories), and slides were counterstained with haematoxylin (incubation for 3 min; Bio-Optica, Milan, Italy).

Images of aortic cross-sections were acquired using an Axioskop Zeiss Imager.M2 microscope (Zeiss) equipped with a digital camera (AxioCam 305 color, Zeiss). The AGTRAP and angiotensinogen signals were quantified using Axiovision Software Rel. 4.7 (Zeiss). The percentage of positive area (intima and media layers) was calculated using the “Area Sum” feature. The ratio of AGTRAP- or angiotensinogen-positive area (brown signal, µm^2^) to total aortic area (µm^2^) was then calculated and multiplied by 100 (% positive signal).

### Western blot analysis

HASMC were lysed in a buffer containing 100 mM Tris (pH 6.8), 20% glycerol, 4% SDS and rat VSMC in RIPA lysis and extraction buffer (Thermofisher Scientific, Waltham, MA, USA) supplemented with protease (Roche, Basel, Switzerland) and phosphatase (Thermofisher Scientific) inhibitors cocktail. Protein concentrations were determined by BCA protein assay kit (Pierce, Rockford, IL, USA). Then dithiothreitol (DTT) 200 mM was added and lysates were boiled for 5 min. Proteins were separated by sodium dodecyl sulfate–polyacrylamide gel electrophoresis (SDS-PAGE) and transferred to nitrocellulose for HASMC or PVDF for rat VSMC membrane by standard procedures. The membranes were blocked with 5% non-fat dry milk powder in 0.05% Tween 20 phosphate-buffered saline (PBS-T) for 1 h. Immunodetection was performed by incubating the membranes with the different primary antibodies for 2 h at room temperature or overnight at 4 °C. After four washes with PBS-T, membranes were incubated with secondary antibody conjugated with horseradish peroxidase (HRP) for 1 h. After four washes, blots were developed with Amersham ECL Plus Western Blotting Detection Reagents and membranes were exposed to ChemiDoc (Bio-Rad, Hercules, CA, USA). Protein levels were evaluated by densitometric analysis using Image Lab Software (Bio-Rad). Protein expression was normalized for β-actin, anti-α-tubulin, or anti-Vinculin protein levels. The following primary antibodies were used to detect the proteins of interest: anti-SIRT1 (H-300, Santa Cruz Biotechnology, Santa Cruz, CA, USA), anti-AGTRAP (ABclonal Technology, Woburn, MA, USA), anti-β-actin (Sigma Aldrich St. Louis, MA, USA), anti-α-tubulin (Calbiochem, San Diego, Ca, USA), and anti-vinculin (Invitrogen, Thermo Fisher Scientific, Waltham, MA, USA).

### Statistical analysis

All data are displayed as the mean with 95% confidence intervals (CI). Each variable was checked for normality distribution by the D’Agostino and Pearson omnibus normality test. Since most of the variables did not pass normality, the difference between two groups was compared either by the two-tailed Mann–Whitney or Wilcoxon rank sum test using GraphPad Prism software (Version 8.0). A *p* < 0.05 was considered statistically significant.

## Results

### Aging and miR-34a expression in the arterial wall and kidney

**Fig. 1:**
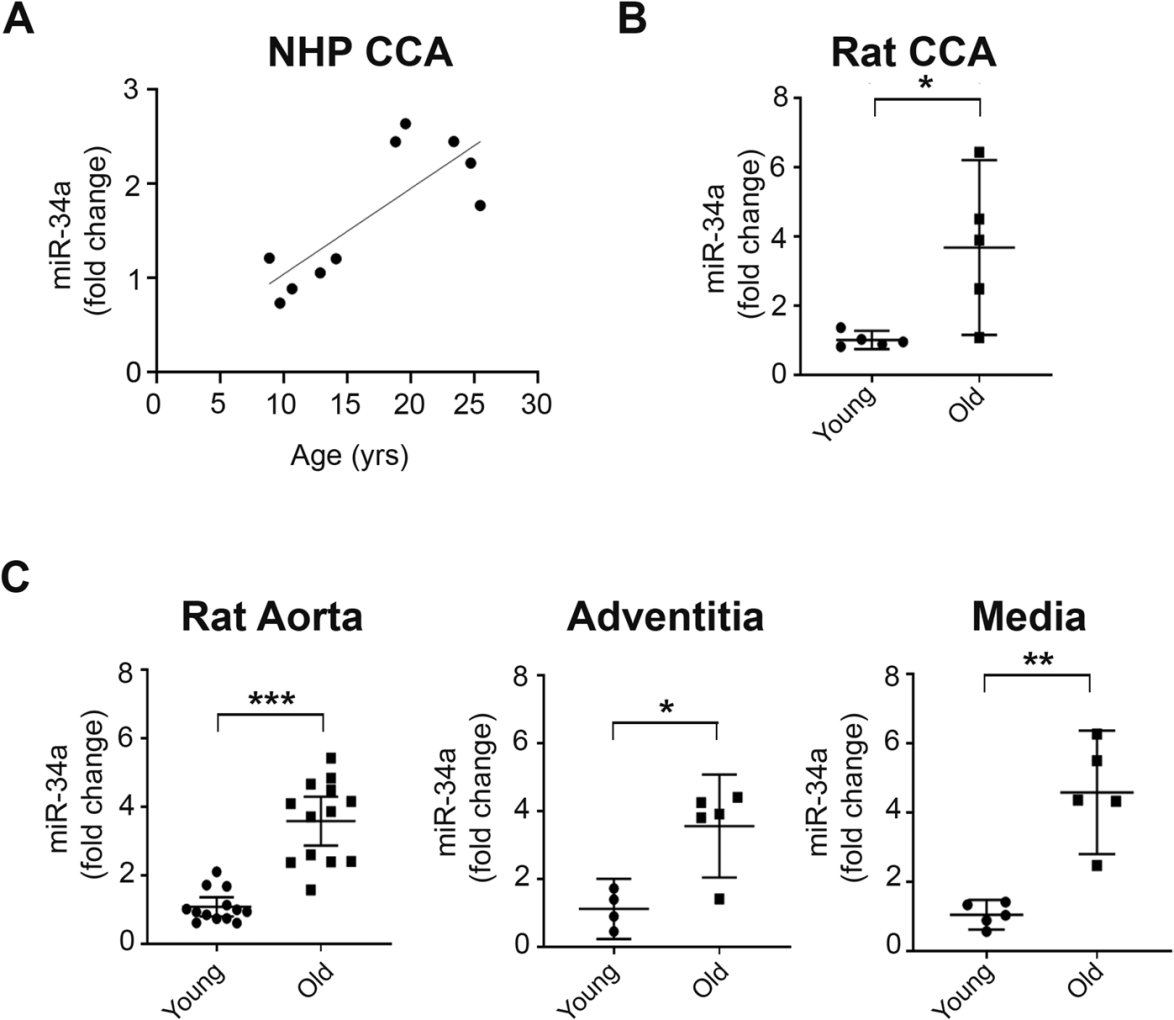
Age-associated increase of miR-34a in monkey and rat central arteries.**A** miR-34a positively correlated with age in the CCA of NHP 8 to 26 years of age (*n* = 10) (*Y* = 0.09097*x* + 0.1310; Rs = 0.6727; *p < 0.05). **B** miR-34a expression was higher in the CCA of 30-month (old) vs 8-month (young) old rats (*n* = 5 in each group). **C** A higher miR-34a expression was also found in the aorta of old vs young rats (*n* = 13 in each group), and this difference was present both in the adventitia (*n* = 4 young, *n* = 5 old) and in the media (*n* = 5 in each group). Statistical analysis was performed using a Mann–Whitney *U* test (*p < 0.05; **p < 0.01; ***p < 0.001)

Our initial aim was to establish if the age-associated increase of miR-34a expression in central arteries, previously shown only in mice [22], also occurs in other mammals. To this end, we examined miR-34a expression in the common carotid artery of Rhesus monkeys of different ages, from 8 to 26 years of age. A linear regression and a positive correlation between miR-34a and age were found (Fig. 1A). Further, we found a ~ 3.5-fold increase in miR-34a expression both in the common carotid artery (Fig. 1B) and in the aorta (Fig. 1C) of 8- vs 30-month-old rats; interestingly, in the aorta, the magnitude of the increase was comparable in the adventitia and media. We also examined Rhesus monkey and rat kidney, an organ that plays a key role in RAAS activation, and found an age-associated increase of miR-34a expression described in NHP by a linear regression and a positive correlation (Suppl. Figure  1 A) and a twofold increase in rat (Suppl. Figure 1B).

These data confirm that miR-34a expression increases with aging in central arteries of NHP and rats and show that, in these species, a similar increase also occurs in the kidney.

### Ang II modulates miR-34a and AGTRAP expression in isolated VSMC

**Fig. 2:**
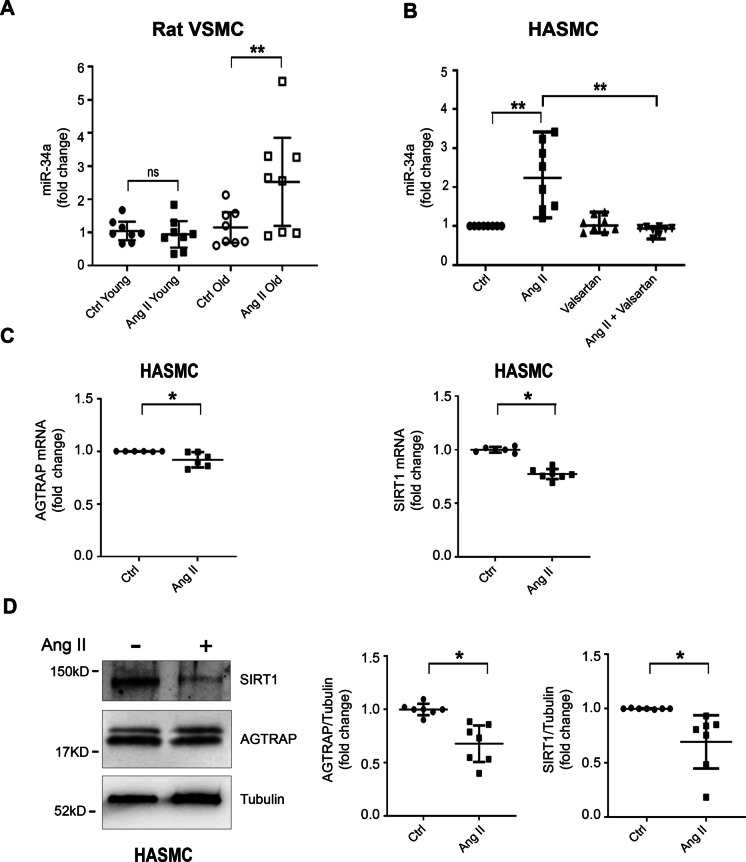
Angiotensin II induces miR-34a upregulation and AGTRAP downmodulation in isolated arterial rat and human aortic smooth muscle cells.**A** VSMC isolated from 8-month (young) and 30-month (old) old rats were treated with Ang II (100 nM for 48 h). Ang II enhanced miR-34a expression in VSMC from old rats but had no effect in VSMC from young rats (*n* = 8 in each group). **B** HASMC (*n* = 6 in each group) were treated with Ang II alone (1 µM for 24 h), or with Valsartan (1 µM for 1 h), followed by treatment with Ang II (1 µM for 24 h). Ang II increased miR-34a expression and this effect was abolished by pretreatment with Valsartan. **C** HASMC was treated with Ang II (1 µM for 24 h). Ang II decreased AGTRAP (*n* = 6 in each group) and SIRT1 mRNA (*n* = 6–7). **D** Representative western blot analysis of AGTRAP and SIRT1 and densitometric ratio of AGTRAP and SIRT1 normalized to tubulin protein levels (*n* = 7 in each group). Ang II decreased both AGTRAP and SIRT1 protein levels. The statistical analysis was performed using the Wilcoxon *t*-test ( *p < 0.05;  **p < 0.01)

The role of Ang II on miR-34a expression is still poorly characterized. We examined the effect of Ang II on miR-34a expression in aortic VSMC isolated from 8- and 30-month-old rats. We found no effect of Ang II on miR-34a expression in young rats; in contrast, miR-34a expression increased ~ 2.5-fold in VSMC of old rats treated with Ang II (Fig. 2A). In additional experiments, Ang II increased miR-34a expression in HASMC, and this effect was abolished by valsartan, an AT1R blocker (Fig. 2B); a response similar to Ang II was obtained upon HASMC exposure to MFGE8, a known relay element within the Ang II signaling cascade (Suppl. Figure 2). Interestingly, Ang II treatment was associated with diminished AGTRAP mRNA (Fig. 2C) and protein (Fig. 2D). It has been previously shown that miR-34a targets SIRT1 and, in agreement with those results [24, 30], we found diminished SIRT1 mRNA (Fig. 2C) and protein (Fig. 2D) associated with the Ang II–induced increase of miR-34a.

### miR-34a and the age-associated decrease in AGTRAP expression in isolated rat aorta VSMC and in mouse aorta

**Fig. 3:**
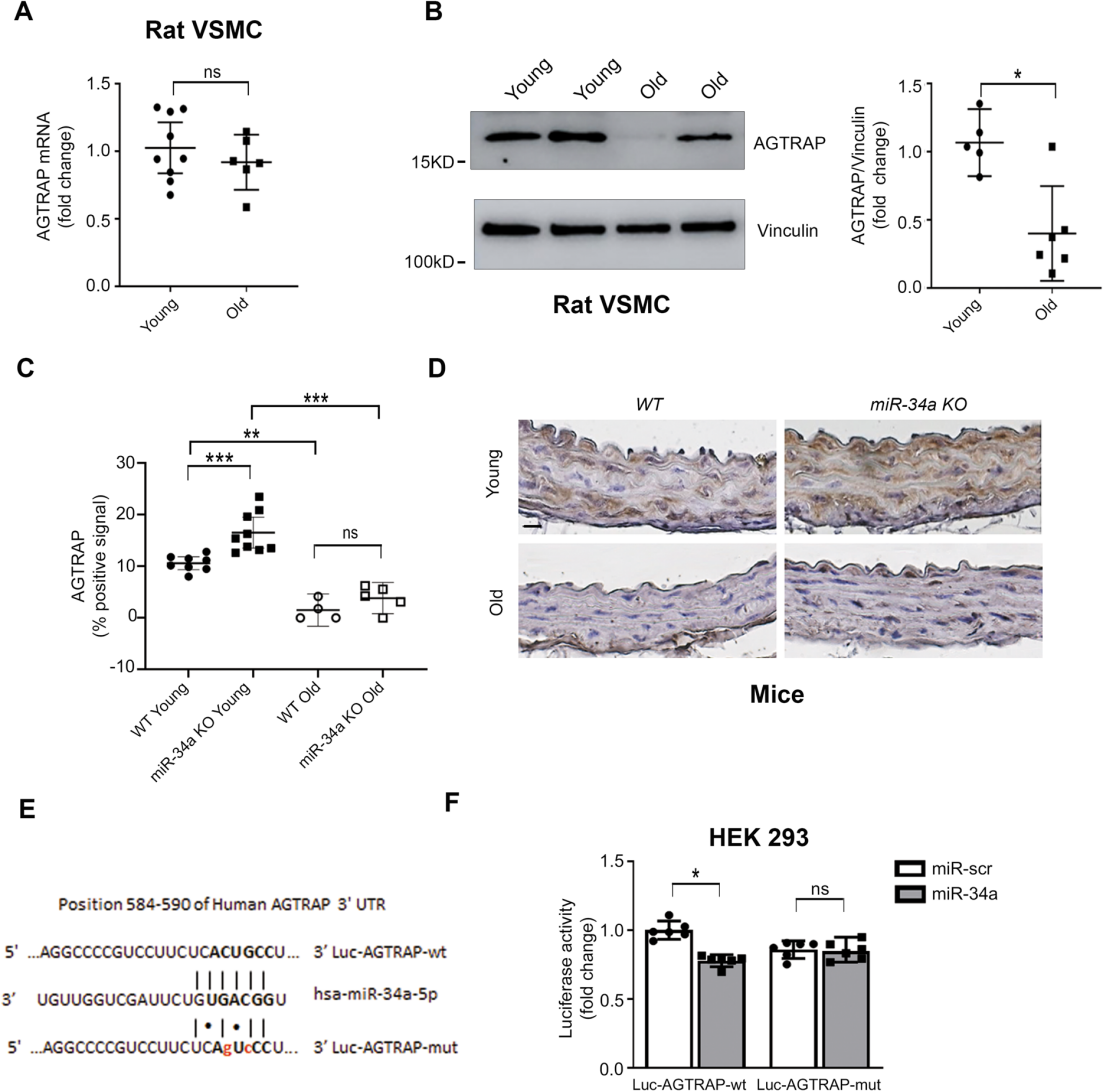
Aging and miR-34a modulate AGTRAP expression. Experiments in (**A–B**) were performed with VSMC isolated from 8-month-old (young) and 30-month-old (old) rats. **A** AGTRAP mRNA expression was not modulated by aging in VSMC of young (*n* = 9) and old (*n* = 6) rats. **B** AGTRAP protein was lower in VSMC of old rats. Representative western blot analysis of AGTRAP in young and old rat VSMC and densitometric ratio of AGTRAP normalized to vinculin protein levels (*n* = 5, young; *n* = 6, old). **C–D** AGTRAP expression was modulated by miR-34a and aging in vivo. Thoracic aortas of WT and miR-34a KO male mice, 2-month-old (young) and 18-month-old (old), were tested for AGTRAP expression by immunohistochemistry. **C** Aging was associated with markedly lower AGTRAP protein expression in both WT and miR-34a KO mice. AGTRAP was higher in young miR-34a KO mice compared to the WT control. In old mice, AGTRAP exhibited a trend toward higher expression in miR-34a KO vs WT, but the difference was not statistically significant. The percentage of positive area per AGTRAP was calculated by determining the ratio of positive AGTRAP area to total aortic cross-sectional area in one section per animal and multiplying by 100. Each circle or square in the graph represents data from a single mouse (WT young, *n* = 8; WT old, *n* = 4; miR-34a KO, young, *n* = 9; miR-34a KO, old, *n* = 5). **D** Representative images (one mouse per group) of the quantification shown in (**C**) of an aortic section field stained for AGTRAP (brown signal) with a specific antibody and counterstained with hematoxylin (purple signal). Calibration bar applied to all panels = 20 µm. **E** AGTRAP is a direct target of miR-34a. HEK293 were transfected with firefly luciferase constructs containing the Human 3′UTR-AGTRAP wild-type (wt) or mutated in miR-34a seed sequence (mut). The panel shows a schematic representation of miR-34a binding sites. The wt and mut seed sequences are indicated in bold, whereas mut nucleotides are indicated in small letters in red. The seed sequence nucleotides indicated are specific for the 3′UTR of Human AGTRAP ENST00000376627.2. **F** These constructs were co-transfected with a plasmid encoding either miR-34a or miR-scramble (miR-scr) sequences as control. Values were normalized according to Renilla luciferase activity (*n* = 6 in each group). The overexpression of miR-34a decreased luciferase activity of 3′UTR-AGTRAP wt but had no effect on 3′UTR-AGTRAP mut. In (**A**), (**B**), and (**C**) the statistical analysis was performed using Mann–Whitney *U* test. In (**F**), statistical analysis was performed using Wilcoxon *t*-test (*p<0.05; **p < 0.01; ***p < 0.001)

We examined AGTRAP expression in aorta VSMC of 8- and 30-month-old rats and found no significant age-associated decrease in AGTRAP mRNA (Fig. 3A); in contrast, AGTRAP protein exhibited a marked age-associated decrease (Fig. 3B).

Further, we examined AGTRAP expression by immunohistochemistry in the aorta of 2- and 18-month-old miR-34a^+/+^ (WT) and miR-34a^−/−^ (KO) mice (Fig. 3C and D). Aging is associated with a markedly decreased AGTRAP expression in the aorta of both old miR-34a KO and WT mice. In young mice, AGTRAP expression was higher in miR-34a KO vs WT mice. In old mice, there was a trend for higher AGTRAP expression in miR-34a KO vs WT mice, but it did not achieve statistical significance. These results suggest that, in addition to miR-34a, other mechanisms contribute to the age-associated decrease of AGTRAP in the arterial wall.

Interestingly, we also found an age-associated increase in mouse aorta VSMC angiotensinogen expression in the aorta of 2- vs 18-month-old mice (Suppl. Figure 3).

To establish a link between miR-34a and AGTRAP expression, we performed in silico analyses of miR-34a potential targets. TargetScan (v8.0) indicated that human AGTRAP is a potential target of miR-34a, suggesting that the age-associated increase in miR-34a in the arterial vascular wall may contribute to decreased AGTRAP expression in arterial VSMC. To this aim, HEK293 cells were transfected with firefly luciferase constructs containing the human 3′UTR-AGTRAP wt or the 3′UTR sequence mutated in the miR-34a seed sequence (3′UTR-AGTRAP mut) (Fig. 3E). These constructs were co-transfected with a plasmid encoding either miR-34a or a miR-scramble sequence. miR-34a overexpression downmodulated the luciferase activity of the 3′UTR wt construct, but not that of the mutated 3′UTR (Fig. 3F). This result indicates that AGTRAP is a direct target of miR-34a.

### Effect of miR-34a overexpression and AGTRAP knockdown on their targets in isolated HASMC

**Fig. 4:**
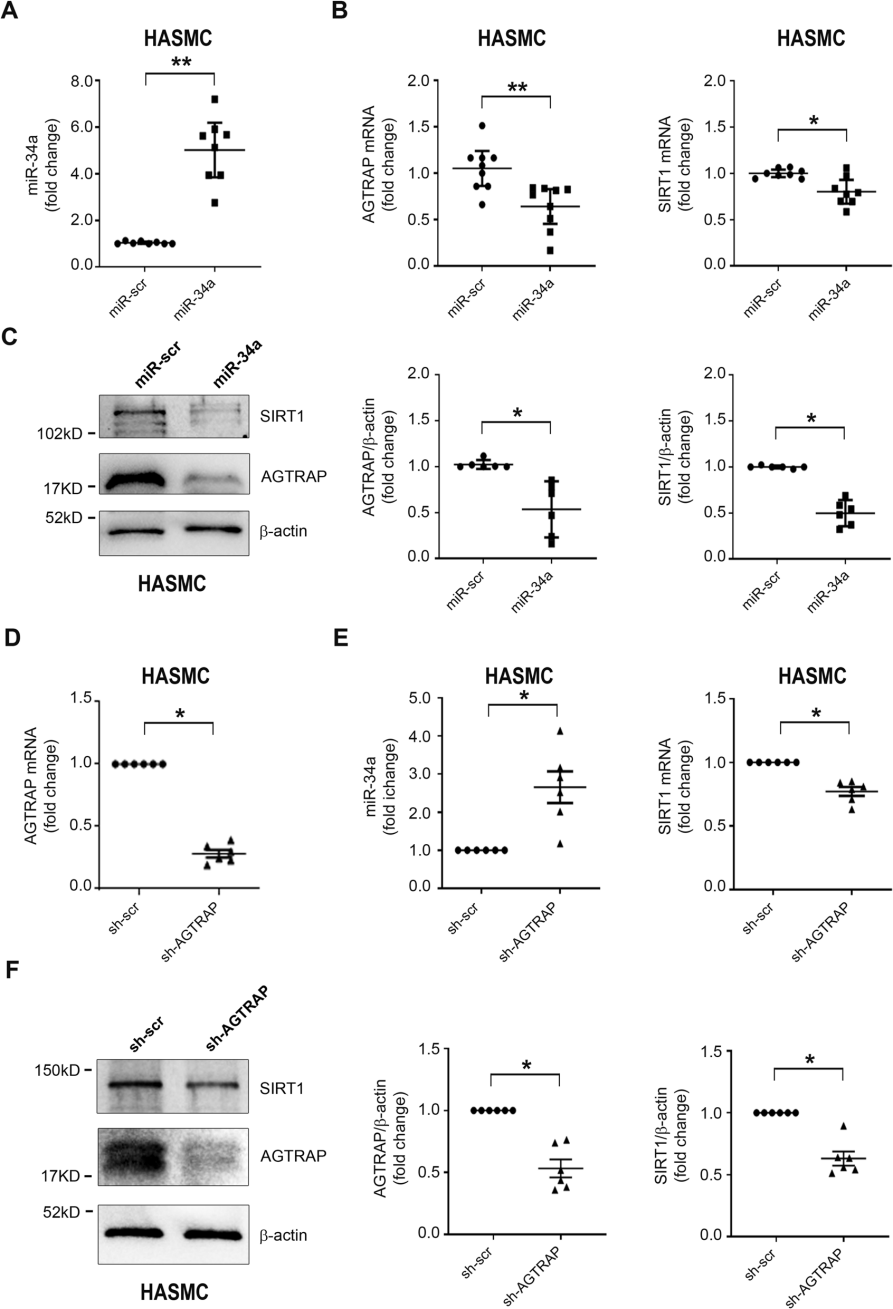
Effect of miR-34a overexpression and AGTRAP knockdown on miR-34a targets in isolated HASMC. **A–C** HASMC were infected for 48 h either with a lentivirus encoding miR-34a or with a control virus (miR-scr); miR-34a (*n* = 8 in each group), AGTRAP (*n* = 9 in each group), and SIRT1 (*n* = 8 in each group). miR-34a and mRNA expression were quantified by RT-qPCR. **A** miR-34a increased, and **B** AGTRAP and SIRT1 mRNA levels were lower in miR-34a overexpressing cells compared to scramble control. **C** Representative western blot analysis and densitometric ratio of AGTRAP and SIRT1, normalized to β-actin protein levels (*n* = 6 in each group), demonstrated a significant decrease of AGTRAP and SIRT1 protein in response to miR-34a forced expression. **D–F** HASMC were infected either with the lentivirus carrying AGTRAP-specific shRNA or with the control virus (sh-scr). AGTRAP (*n* = 6 in each group), miR-34a (*n* = 6 in each group), and SIRT1 (*n* = 6 in each group). miR-34a and mRNA expression were quantified by RT-qPCR. **D** shAGTRAP decreased AGTRAP mRNA. **E** Upon AGTRAP knockdown, miR-34a increased, whereas SIRT1 mRNA decreased. **F** Representative western blot analysis and densitometric ratio demonstrate that AGTRAP and SIRT1 protein expression levels decreased upon AGTRAP knockdown. Statistical analysis was performed using the Wilcoxon *t-*test (*p < 0.05; **p < 0.01)

We then examined the effect of miR-34a on AGTRAP expression. HASMC were infected either with a lentivirus encoding miR-34a or a control virus (miR-scr), then cells were analyzed for miR-34a (Fig. 4A), AGTRAP, and SIRT1mRNA expression (Fig. 4B) by RT-qPCR. The forced expression of miR-34a in HASMC decreased both AGTRAP and SIRT1 mRNA (Fig. 4B). AGTRAP and SIRT1 proteins were evaluated by western blotting analysis, and both AGTRAP and SIRT1 decreased upon miR-34a overexpression (Fig. 4C).

Altogether, our results show that Ang II induces miR-34a and that miR-34a directly targets AGTRAP, downmodulating its expression. AGTRAP is known to inhibit Ang II effects mediated by AT1R; therefore, its downmodulation would be expected to enhance miR-34a via increased Ang II signaling. In contrast, AGTRAP upregulation would be expected to inhibit miR-34a expression. We then evaluated the possibility that AGTRAP may modulate miR-34a expression, as well as the mRNA and protein levels of SIRT1, a well-known miR-34a target.

We first examined the effect of AGTRAP downmodulation. To this aim, HASMC were infected either with a lentivirus carrying AGTRAP-specific shRNA (sh-AGTRAP) or with the control virus (sh-scr). As expected, sh-AGTRAP knocked down AGTRAP mRNA (Fig. 4D) and protein (Fig. 4F), and this effect of sh-AGTRAP was associated with enhanced expression of miR-34a (Fig. 4E). Moreover, in agreement with the results shown above (Fig. 4B and C), the increased expression of miR-34a lowered SIRT1 mRNA (Fig. 4E) and protein (Fig. 4F).

### Negative feedback loop between AGTRAP and miR-34a in isolated HASMC

**Fig. 5:**
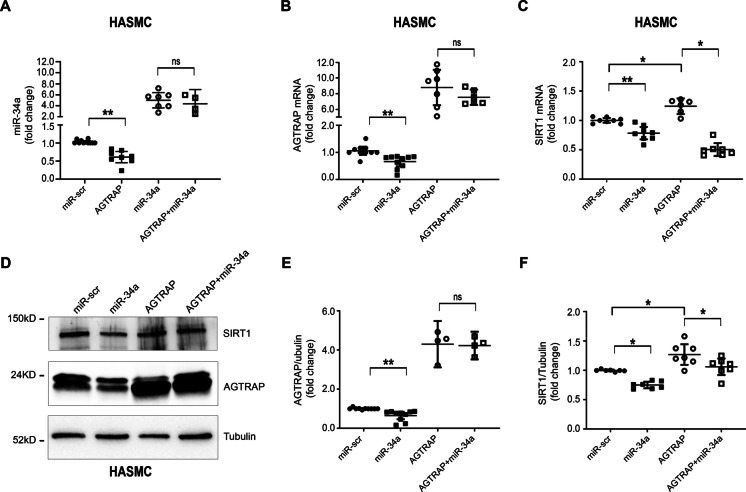
A negative feedback loop exists between AGTRAP and miR-34a in isolated HASMC. HASMC were co-infected with lentiviruses encoding AGTRAP and miR-34a. Single infection either with AGTRAP or miR-34a was performed together with miR-scramble virus or AGTRAP backbone vector, respectively. **A** AGTRAP overexpression significantly decreased miR-34a expression under baseline conditions but not when miR-34a was overexpressed (miR-scr, *n* = 11; AGTRAP, *n* = 8; miR-34a, *n* = 7; AGTRAP + miR-34a, *n* = 4). **B** AGTRAP mRNA diminished in response to miR-34a, and this effect was abolished when both miR-34a and AGTRAP were overexpressed (miR-scr, *n* = 12; miR-34a, *n* = 10; AGTRAP, *n* = 7; AGTRAP + miR-34a, *n* = 6); **C** SIRT1 mRNA diminished in response to miR-34a overexpression both under baseline conditions and when both miR-34a and AGTRAP were co-infected (miR-scr, *n* = 8; miR-34a, *n* = 8; AGTRAP, *n* = 6; AGTRAP + miR-34a, *n* = 7). **D** Representative Western blot analysis of SIRT1 and AGTRAP protein expression levels in HASMC. **E–F** Densitometric ratio of AGTRAP and SIRT1, respectively, normalized to tubulin protein levels; **E** AGTRAP forced expression abolished the effect of miR-34a to decrease AGTRAP protein (miR-scr, *n* = 10; miR-34a, *n* = 10; AGTRAP, *n* = 4; AGTRAP + miR-34a, *n* = 4). **F** SIRT1 protein decreased in response to miR-34a forced expression and increased when AGTRAP was overexpressed. The effect of AGTRAP to downmodulate SIRT1 persisted when both AGTRAP and miR-34a were co-infected (*n* = 7 in each group). Statistical analysis was performed using the Wilcoxon *t-*test (*p < 0.05; **p < 0.01)

We then examined the effect of AGTRAP overexpression. HASMC were infected either with lentiviruses encoding an AGTRAP allele devoid of the 3′UTR, thus lacking the miR-34a target sequence, or encoding miR-34a, or co-infected with both lentiviruses. AGTRAP overexpression decreased miR-34a under baseline conditions and had no significant effect in the presence of miR-34a forced expression (Fig. 5A). Further, miR-34a overexpression decreased AGTRAP mRNA and protein under baseline conditions but had no effect in the presence of AGTRAP forced expression (Fig. 5B, D, and E). The cross-modulation of miR-34a and AGTRAP yielded the expected changes in SIRT1; miR-34a overexpression decreased SIRT1 mRNA (Fig. 5C) and protein (Fig. 5D and F) under baseline conditions and, in agreement with the direct effect of miR-34a on SIRT1 expression, this effect persisted when miR-34a was co-expressed with AGTRAP (Fig. 5C, D, and F). Interestingly, the forced expression of AGTRAP alone increased SIRT1 mRNA and protein (Fig. 5C and F).

Altogether, these experiments demonstrate the negative feedback loop existing between miR-34a and AGTRAP and their role in SIRT1 modulation and, possibly, in other miR-34a targets.

### AGTRAP inhibits the pro-inflammatory action of angiotensin II and miR-34a in isolated HASMC

**Fig. 6:**
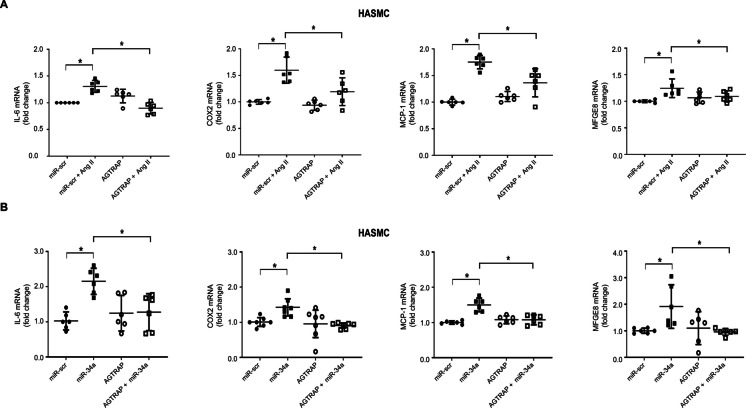
AGTRAP forced expression decreases inflammation induced by Angiotensin II and miR-34a in isolated HASMC. **A** HASMC was treated with Ang II (1 µM for 24 h). Total RNA was extracted and assayed for IL-6, COX2, MCP-1, and MFG-E8 by RT-qPCR. Expression levels of IL-6 (Ctrl, *n* = 6; Ang II, *n* = 8), COX2 (*n* = 6 in each group), MCP-1 (*n* = 6 in each group), and MFGE8 (*n* = 6 in each group) mRNA increased in response to Ang II, and this effect was either abolished or markedly inhibited in cells overexpressing AGTRAP. **B** HASMC were single-infected with lentiviruses encoding control scramble (miR-scr), miR-34a, AGTRAP, and double-infected with miR-34a and AGTRAP. Total RNA was extracted and assayed for IL-6, COX2, MCP-1, and MFGE8 by RT-qPCR. miR-34a overexpression increased IL-6 (*n* = 6), COX2 (*n* = 7), MCP-1 (*n* = 6), and MFGE8 (*n* = 6) mRNA expression; under these conditions, simultaneous expression of AGTRAP abolished the pro-inflammatory action of miR-34a and restored the baseline expression of these genes. Statistical analysis was performed using the Wilcoxon *t-*test (*p < 0.05)

In agreement with prior studies [8, 30–32], we found that Ang II induced the expression of IL-6, COX2, MCP-1, and MFGE8 mRNA in HASMC (Fig. 6A); this pro-inflammatory effect of Ang II was either abolished or inhibited by AGTRAP forced expression, likely because of enhanced AT1R internalization.

A pro-inflammatory effect of miR-34a in VSMC has been previously described [22, 33], but the mechanisms underlying this response to miR-34a are still poorly characterized. We found that the effect of Ang II to upregulate IL-6, COX2, MCP-1, and MFGE8 mRNA is mimicked by miR-34a overexpression (Fig. 6B). Under these conditions, AGTRAP forced expression had no effect per se on IL-6, COX2, MCP-1, and MFGE8 mRNA but abolished the effect of miR-34a to increase the expression of the mRNAs of these pro-inflammatory cytokines.

Taken together, these experiments show that miR-34a downmodulates AGTRAP and induces the expression of inflammatory genes; in contrast, AGTRAP overexpression downmodulates the pro-inflammatory effect of Ang II and miR-34a.

## Discussion

The focus of the present study was to elucidate the crosstalk between miR-34a and AGTRAP, an inhibitor of AT1R signaling, and the effects of this crosstalk on Ang II downstream responses mediated by AT1R.

We used different experimental models, as required by the specific questions to be addressed. The effect of aging on miR-34a expression in central arteries was evaluated both in rats and in NHP, the animal species closest to man. The effect of miR-34a and aging on AGTRAP expression was shown in young and old mice, WT and KO for miR-34a. Mechanistic experiments were performed in vitro in rat aorta VSMC isolated from young and old rats and in human aorta smooth muscle cells.

The major findings of this work are: (i) both Ang II and MFGE8 increase miR-34a expression in VSMC; (ii) AGTRAP is a direct target of miR-34a; (iii) Ang II decreases AGTRAP mRNA and protein in HASMC; (iv) miR-34a and AGTRAP cross-modulate their expression via a negative feedback loop, with the expected changes in Ang II signaling; (v) AGTRAP protein decreases with aging in mouse aorta and in rat VSMC.

### miR-34a expression and arterial aging

It has been previously shown that miR-34a expression increases with aging in a variety of tissues including the heart [19, 34], brain [35], liver [36], endothelium, and spleen [37]; and our results demonstrate an age-associated increase of miR-34a expression also in the kidney. However, miR-34a expression in arterial aging has been addressed only by two original studies by one of the authors of this paper (A.R.); it has been shown that miR-34a expression is higher in the aorta of 21-month-old vs 2.5-month-old mice [22] and exhibits an age-associated increase in HASMC obtained from 3 men within a very limited age range (22, 30, and 43 years of age at p5 and p15) [33]. The present study extends those previous findings by showing an age-associated increase of miR-34a expression in rat CCA and aorta, and in NHP CCA. The mechanisms for the age-associated increase in miR-34a are still poorly characterized. It is known that an increase in oxidative stress, which is considered a key feature of aging since Harman first proposed the free radical theory of aging [38], and is widely regarded as a mechanism of aging [39], enhances miR-34a expression [40]. In this study, we show evidence that Ang II upregulates miR-34a in VSMC isolated from the aorta of 30-month-old rats and in HASMC. Interestingly, we also found that MFGE8, a pro-inflammatory molecule positively modulated by aging and Ang II, highly expressed in arteries of old subjects [7, 8], increases miR-34a expression in HASMC.

Ang II is known to induce pro-inflammatory cytokines that contribute to the development of the SASP [31] and to the low-level “sterile” inflammation associated with aging; further, the forced expression of miR-34a in VSMC mimics the effect of Ang II and induces the expression of inflammatory molecules [22, 33]. We confirmed the pro-inflammatory effect of both Ang II and miR-34a in VSMC, and we showed that Ang II and miR-34a’s ability to increase IL-6, COX2, MCP-1, and MFGE8 is markedly inhibited by the forced expression of AGTRAP.

### AGTRAP expression and arterial aging

AGTRAP was initially described by Dzau [11] as an AT1R-associated protein that inhibits Ang II signaling via this receptor by inducing its internalization [11–13]; numerous subsequent studies have examined AGTRAP’s role in a variety of cardiovascular and renal diseases associated with enhanced RAAS activation and Ang II signaling. However, no prior study has identified miR-34a as the link between Ang II signaling and AGTRAP. Moreover, SIRT1 protein, an important miR-34a target, decreases in response to AGTRAP knockdown, and it is known that the decrease in SIRT1 enhances AT1R expression [41], linking the decrease of SIRT1 to the increase in Ang II signaling.

Our results clearly show that AGTRAP protein decreases in VSMC isolated from old rats and in the aorta of old mice. In agreement with our result showing that AGTRAP is a direct miR-34a target, AGTRAP expression is higher in the aorta of 2-month-old miR-34a KO vs WT mice. However, AGTRAP expression is markedly diminished, both in 18-month-old mice WT and miR-34a KO and, although there is a trend for higher AGTRAP expression in miR-34a KO vs WT old mice, the difference did not achieve statistical significance. This result suggests that other mechanisms besides miR-34a determine the age-associated decrease of AGTRAP expression in the arterial wall The absence of a significant difference in AGTRAP expression in miR-34a KO vs WT old mice could be related to the low number of old miR-34a KO and WT mice examined in our study, 5 and 4 respectively, possibly too low to achieve sufficient statistical power and to demonstrate a difference between the two groups. Unfortunately, no other old mice were available in our colony, and increasing the number of animals in each group would have required a significant time delay and was therefore not feasible. Future studies will be needed to fully elucidate the molecular mechanism underlying the age-associated decrease of AGTRAP in central arteries in WT and miR-34a KO mice.

Interestingly, we also found an age-associated increase of angiotensinogen expression in mouse aorta VSMC , another mechanism that may contribute to RAAS activation in the elderly.

It is noteworthy that age-associated remodeling of the central arteries is a condition largely driven by enhanced Ang II signaling and is characterized by intima-media thickening, a substrate that favors the development of atherosclerosis and central artery stiffening, a major cause of heart failure in the elderly [1, 2]. The age-associated decrease in AGTRAP demonstrated in the present work enhances the effects of Ang II mediated by AT1R. In agreement with these findings, AGTRAP overexpression inhibits Ang II–induced DNA synthesis [13] and Ang II–induced oxidative stress and senescence in isolated VSMC [15]. Further, AGTRAP overexpression in vivo lowers blood pressure in angiotensin-dependent [17] and high salt-induced hypertension [42], and AGTRAP KO is associated with increased renal fibrosis and a shorter life span [16].

It is well established that Ang II [43], as well as other agonists (such as endothelin-1 [44], IGF-1 [45], phenylephrine [46], and PDGF [47]) induce phosphoinositide turnover, leading to the formation of inositol 1,4,5-trisphosphate (IP3) that releases Ca^2+^ from an intracellular store [43, 48]. The IP3-triggered increase in cytosolic [Ca^2+^] (Ca_i_) causes VSMC contraction, leading to hypertension in vivo [49].

In addition to promoting AT1R internalization, AGTRAP has been shown to associate with sarco/endoplasmic reticulum Ca^2+^-ATPase (SERCA)2a and to enhance its activity in cardiomyocytes. AGTRAP KO impairs Ca_i_ uptake into the sarcoplasmic reticulum, leading to altered Ca_i_ homeostasis and Ca^2+^ depletion of an intracellular store [50, 51]. In isolated myocardial cells, AGTRAP deletion is associated with a delayed and prolonged Ca_i_ transient and, as a consequence, with delayed time to peak contraction and relaxation [51], two major features of myocardial contraction in aging [1, 2]. These functional effects in single myocardial cells are paralleled by delayed relaxation, i.e., diastolic dysfunction, in the mouse heart, in vivo [51]. Since the age-associated increase of miR-34a also occurs in the heart [34], the findings of the present study raise the possibility that miR-34a-dependent AGTRAP down-modulation may underlie diastolic dysfunction and heart failure with preserved ejection fraction, the most common form of heart failure in the elderly population.

It is still unknown whether AGTRAP associates with SERCA2a and, possibly, with SERCA2b, the other SERCA isoform present in VSMC, and enhances SERCA activity in VSMC, as it does in myocardial cells. Nevertheless, it has been shown that chronic increase of Ca_i_ concentration, as it occurs in the presence of dysfunctional Ca^2+^ handling proteins such as SERCA2a, enhances VSMC proliferation, a prerequisite for neointima accumulation and increased intima-media thickness in aging. The restoration of effective Ca_i_ uptake by sarco/endoplasmic reticulum inhibits VSMC proliferation both in vitro and in vivo [52, 53].

Therefore, it is likely that the age-associated decrease of AGTRAP expression induces VSMC aging phenotype via two synergic but independent mechanisms: (i) the enhancement of AT1 receptor signaling and (ii) the decreased Ca_i_ uptake by the sarco/endoplasmic reticulum and Ca^2+^ depletion of this intracellular store.

### Limitations of the present study

This work has some limitations that should be addressed in future studies.

We have not examined the ability of forced AGTRAP expression to prevent arterial aging in vivo. This question could have been addressed by establishing the effect of aging on central arteries of AGTRAP transgenic mice, but such a project was beyond the time frame of the present work.

Future studies with larger animal groups will be needed to establish if there is a significant difference in AGTRAP protein expression in the aortic wall of old miR-34a WT and KO mice. Our experiments were limited by the relatively small number of old mice in each group.

The mechanisms underlying the decrease of AGTRAP expression observed in old miR-34a KO mice remain unclear and should be investigated in future studies.

## Conclusions

Similarly to AT1R and Ang II, AGTRAP is expressed in many tissues, and the findings of the present study, although limited to arteries and arterial smooth muscle cells, are relevant to other cell types and pathologic conditions. Within the cardiovascular field, RAAS activation associated with enhanced Ang II signaling and the consequent pro-inflammatory and pro-fibrotic response occurs in a variety of diseases, including heart failure, cardiac hypertrophy, diabetic cardiomyopathy, hypertension, and atrial fibrillation [54]. Moreover, considering our findings, the response to ACE inhibitors and AT1R blockers, commonly used drugs in the treatment of important cardiovascular diseases, would be expected to decrease miR-34a expression, leading to an increase in AGTRAP and further inhibition of AT1R signaling and of the downstream effects of Ang II; this represents a previously undescribed mechanism of action of  ACE inhibitors and AT1R blockers.

Altogether, the results of the present work identify a novel negative feedback loop between miR-34a and AGTRAP, which represents a new mechanism to reinforce Ang II signaling via AT1R in pathologic conditions associated with RAAS activation.

## Supplementary Information

Below is the link to the electronic supplementary material.

ESM 1 **Age-associated increase of miR-34a in monkey and rat kidney. (A)** miR-34a positively correlated with age in kidney of NHP from 5.8 to 30.5 years of age (n=20) (Y=0.0537x + 0.489, Rs=0.6917; ***p<0.001). **(B)** miR-34a expression was higher in the kidney of 30 months old (Old) vs 8 months old (Young) rats (n=9 Young, n=8 Old). Statistical analysis was performed using a Mann Whitney *U* Test (***p<0.001). (PNG 31.3 KB)

ESM 1 (TIF 123 KB)

ESM 2 **MFGE8 increases miR-34a expression in isolated HASMCs.** HASMCs were treated with MFGE8 (100 ng/ml for 48 h). miR-34a expression increased in MFGE8-treated HASMCs compared to the control group (n=6 in each group). Statistical analysis was performed using a Wilcoxon *t-*test ( *p<0.05). (PNG 13.6 KB)

ESM 2 (TIF 61.4 KB)

ESM 3 **Angiotensinogen expression is modulated by aging in mouse aorta.** Thoracic aortas of 2 months (Young; n=5) and 18 months (Old; n=4) mice were tested for angiotensinogen expression by immunohistochemistry. Aging was associated with higher angiotensinogen protein expression. **(A)** Quantification of angiotensinogen positive area (%). Each circle in the graph represents data from a single mouse. Statistical analysis was performed using a Mann Whitney *U* Test (*p<0.05). **(B)** Representative images (one mouse per group) of the quantification shown in panel A of an aortic section field stained for angiotensinogen (brown signal) with a specific antibody and counterstained with hematoxylin (purple signal). Calibration bar applies to all panels =20 μm. (PNG 266 KB)

ESM 3 (TIF 886 KB)

## Data Availability

The data, analytic methods, and study materials will be made available from the corresponding author upon reasonable request to other researchers for the purpose of reproducing the results or replicating the procedure.

## Abbreviations

ACE: Angiotensin-converting enzyme
ACE II: Angiotensin-converting enzyme II
Ang I: Angiotensin I
Ang II: Angiotensin II
Ang (1–7): Angiotensin 1–7
AT1R: Angiotensin II type-1 receptor
AT2R: Angiotensin II type-2 receptor
ATRAP/Agtrap: Angiotensin II type-1 receptor-associated protein
COX2: Cyclooxygenase-2
Ca_i_: Cytosolic [Ca^2+^]
HASMC: Human aortic smooth muscle cells
IL-6: Interleukin-6
IP3: Inositol 1,4,5-trisphosphate
IP3R: IP3 receptor
KO: Knockout
MCP-1: Monocyte chemoattractant protein-1
MFGE8: Milk fat globule-epidermal growth factor 8
miRNAs: MicroRNAs
miR-34a: MicroRNA-34a
RAAS: Renin–angiotensin–aldosterone system
SIRT1: Sirtuin 1
SERCA2a: Sarco/endoplasmic reticulum Ca^2+^-ATPase 2a
SR/ER: Sarcoplasmic reticulum/endoplasmic reticulum
VSMC: Vascular smooth muscle cells
WT: Wild type
