# Supplementary figures and images for: Angiotensin II, miR-34a, and AGTRAP crosstalk in arterial smooth muscle cells
Source: GeroScience. 2025 Nov 26;48(1):197–215. doi: 10.1007/s11357-025-02018-5 (PMC12972272; doi:10.1007/s11357-025-02018-5)

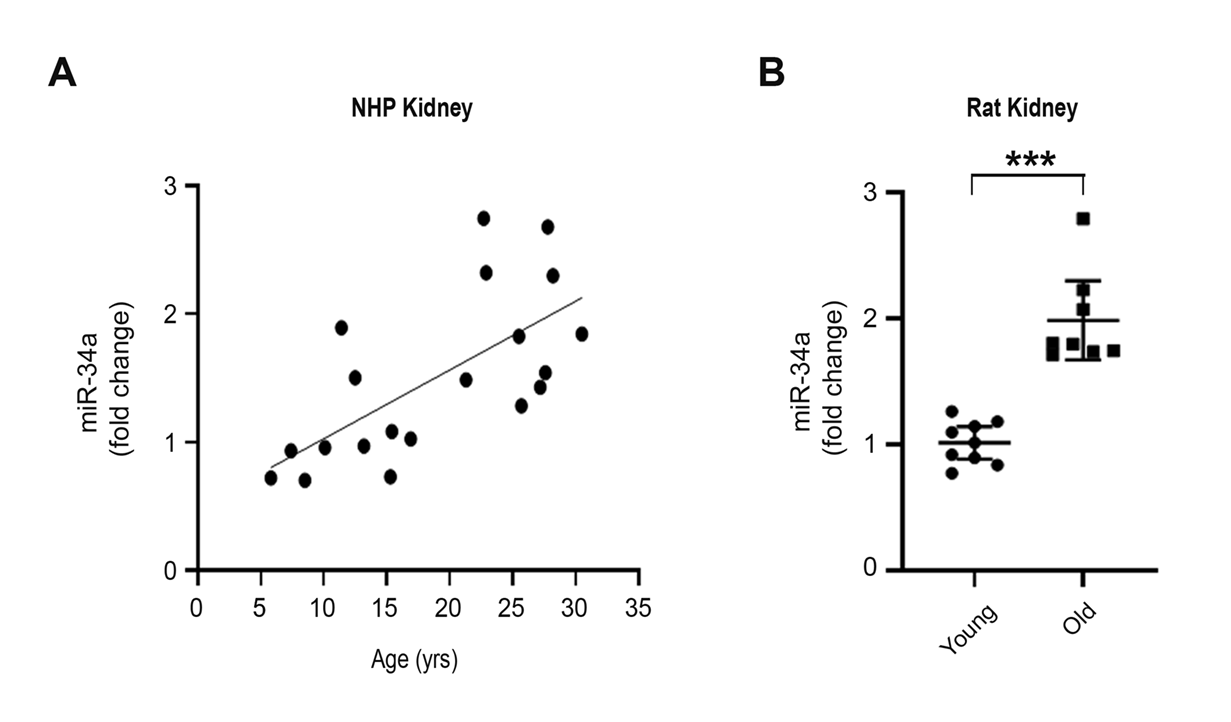

Supplement: Supplementary file 1 — Age-associated increase of miR-34a in monkey and rat kidney. (A) miR-34a positively correlated with age in kidney of NHP from 5.8 to 30.5 years of age (n=20) (Y=0.0537x + 0.489, Rs=0.6917; ***p<0.001). (B) miR-34a expression was higher in the kidney of 30 months old (Old) vs 8 months old (Young) rats (n=9 Young, n=8 Old). Statistical analysis was performed using a Mann Whitney U Test (***p<0.001). (PNG 31.3 KB) [file 11357_2025_2018_Fig7_ESM.png]

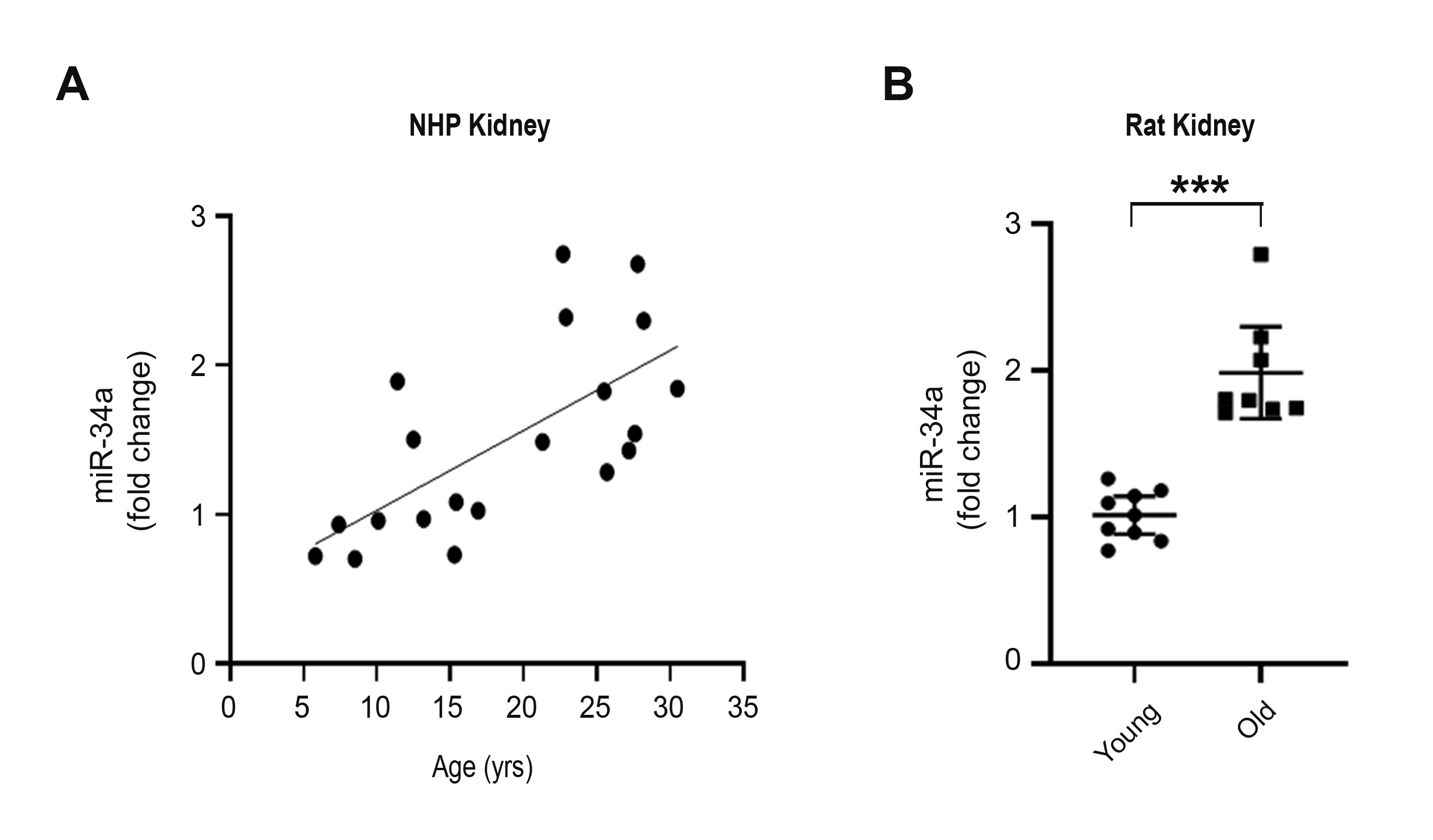

Supplement: Supplementary file 2 — (TIF 123 KB) [file 11357_2025_2018_MOESM1_ESM.tif]

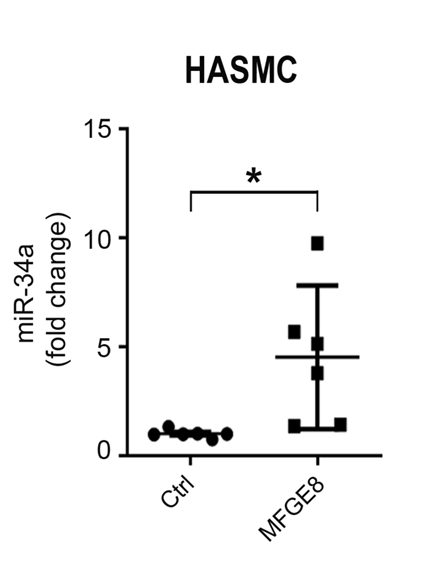

Supplement: Supplementary file 3 — MFGE8 increases miR-34a expression in isolated HASMCs. HASMCs were treated with MFGE8 (100 ng/ml for 48 h). miR-34a expression increased in MFGE8-treated HASMCs compared to the control group (n=6 in each group). Statistical analysis was performed using a Wilcoxon t-test ( *p<0.05). (PNG 13.6 KB) [file 11357_2025_2018_Fig8_ESM.png]

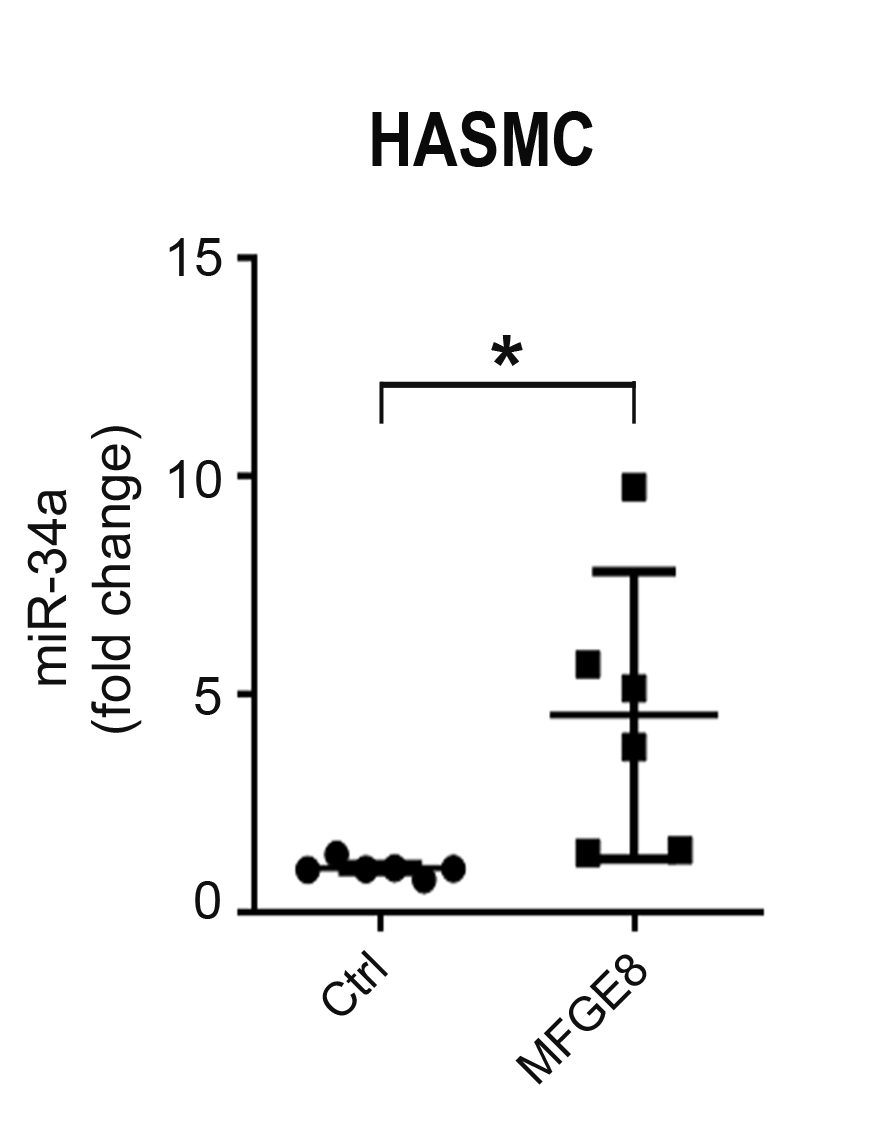

Supplement: Supplementary file 4 — (TIF 61.4 KB) [file 11357_2025_2018_MOESM2_ESM.tif]

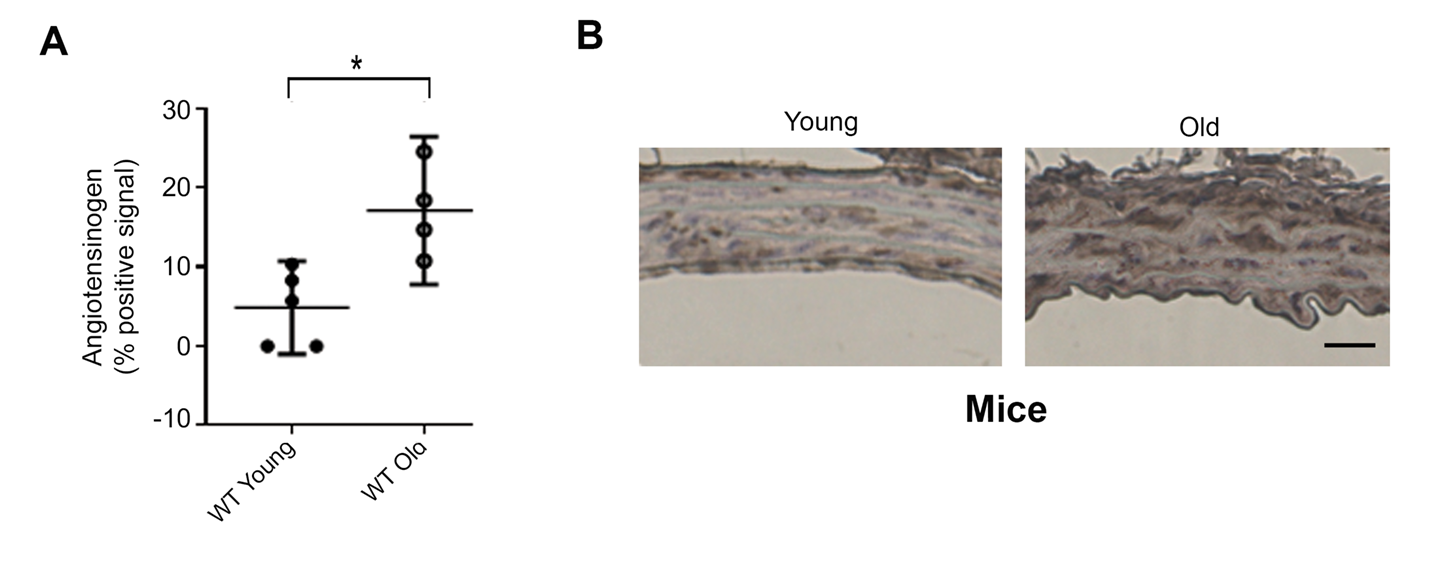

Supplement: Supplementary file 5 — Angiotensinogen expression is modulated by aging in mouse aorta. Thoracic aortas of 2 months (Young; n=5) and 18 months (Old; n=4) mice were tested for angiotensinogen expression by immunohistochemistry. Aging was associated with higher angiotensinogen protein expression. (A) Quantification of angiotensinogen positive area (%). Each circle in the graph represents data from a single mouse. Statistical analysis was performed using a Mann Whitney U Test (*p<0.05). (B) Representative images (one mouse per group) of the quantification shown in panel A of an aortic section field stained for angiotensinogen (brown signal) with a specific antibody and counterstained with hematoxylin (purple signal). Calibration bar applies to all panels =20 μm. (PNG 266 KB) [file 11357_2025_2018_Fig9_ESM.png]

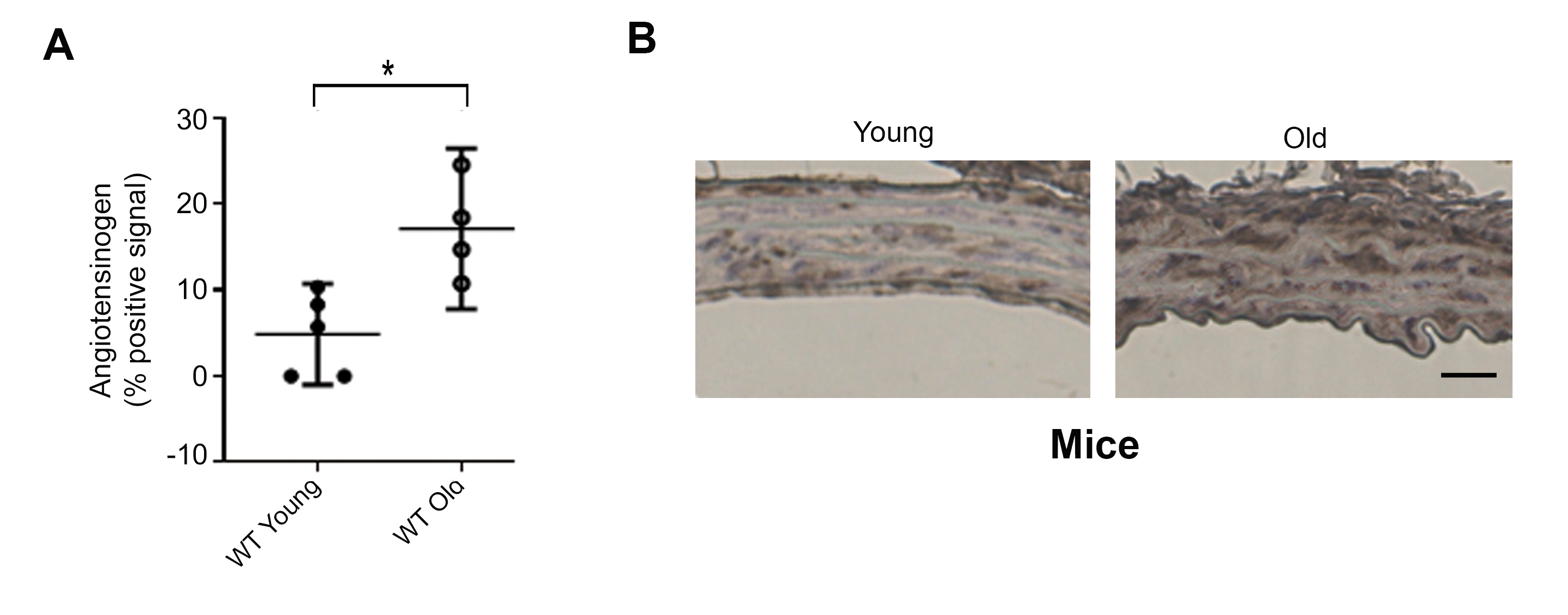

Supplement: Supplementary file 6 — (TIF 886 KB) [file 11357_2025_2018_MOESM3_ESM.tif]
